# *In situ* SERS detection of dissolved nitrate on hydrated gold substrates†

**DOI:** 10.1039/d1na00156f

**Published:** 2021-06-12

**Authors:** Timo Küster, Geoffrey D. Bothun

**Affiliations:** Department of Chemical Engineering, University of Rhode Island 2 East Alumni Ave, Kingston RI 02881 USA gbothun@uri.edu +1-401-874-9518

## Abstract

The accurate and fast measurement of nitrate in seawater is important for monitoring and controlling water quality to prevent ecologic and economic disasters. In this work we show that the *in situ* detection of nitrate in aqueous solution is feasible at nanomolar concentrations through surface enhanced Raman spectroscopy (SERS) using native nanostructured gold substrates without surface functionalization. Spectra were analyzed as collected or after standard normal variate (SNV) normalization, which was shown through Principal Component Analysis (PCA) to reduce spectral variations between sample sets and improve Langmuir adsorption model fits. An additional normalization approach based on the substrate silicon template showed that silicon provided an internal standard that accounted for the spectral variance without the need for SNV normalization. Nitrate adsorption was well-described by the Langmuir adsorption model, consistent with an adsorbed monolayer, and a limit of detection of 64 nM nitrate was obtained in ultrapure water, representing environmentally relevant concentrations. Free energy calculations based on the Langmuir adsorption constants, approximating equilibrium adsorption constants, and calculated self-energy arising from image charge, accounting for electrostatic interactions with a polarizable nanostructured substrate, suggest that nitrate adsorption was partially driven by an entropy gain presumably due to dehydration of the gold substrate and/or nitrate ion. This work is being extended to determine if similar statistical and normalization methods can be applied to nitrate detection in complex natural waters where non-target ions and molecules are expected to interfere.

## Abstract 2

SERS spectral processing and statistical analysis enable the ultrasensitive *in situ* detection of nitrate ion on nanostructured gold.

## Graphical abstract

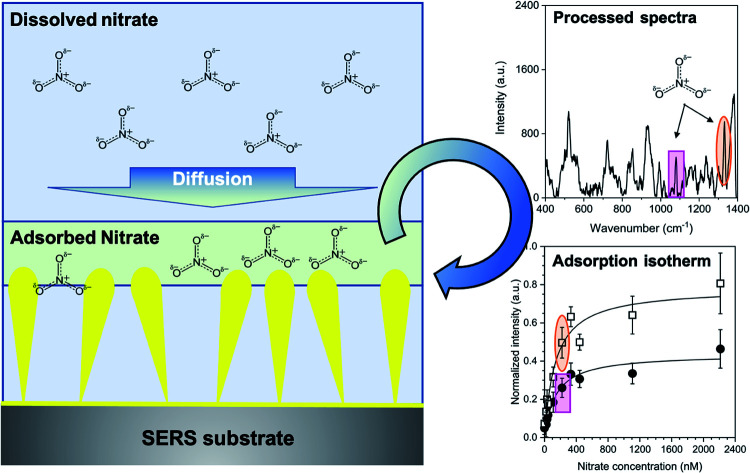

## Introduction

The ocean is habitat for millions of species and plays a key role in controlling the climate by storing greenhouse gases and acting as a heat sink. It has been utilized by humans for centuries to provide food, transportation and economic growth.^1,2^ However, human activity, such as the use of fertilizers, discharge from wastewater treatment facilities and the use of combustion fuels can lead to elevated levels of nitrogen in the ocean. Nitrogen is an essential part of the nutrient cycle and enables marine life through primary production.^2–5^

If nitrate and other nutrients reach excessive levels they can contribute to eutrophication, which is a process where rapid plant growth diminishes the supply of dissolved oxygen necessary to support higher trophic marine life. In addition to creating “dead zones” for respiring species, eutrophication can promote algae growth and the formation of large algae blooms that release toxins such as domoic acid into the surrounding waters.^6,7^ The negative economic impacts of eutrophication tied to fishing, aquaculture, and tourism can be significant, as can be the adverse health effects of individuals who are exposed to harmful algae blooms (HABs). Affordable, deployable, and accurate tools to improving the spatiotemporal detection of inorganic nitrogen *in situ* will increase ecological monitoring and inform computational approaches that are being developed to predict emerging conditions that may lead to the formation of HABs. Early warning of these devastating ecological events will provide additional time to alert coastal stakeholders and enact countermeasures.

Many methods exist for the detection of nitrate in fresh and wastewater.^8^ However, the continuous in-field detection of nitrate in seawater is less explored. The most commonly used method for nitrate detection in seawater is UV-vis spectroscopy with, for example, commercially available instruments providing limits of detection as low as 0.5 μM.^9^

Surface Enhanced Raman Spectroscopy (SERS) provides an ultrasensitive platform for sensor design and has been described as a molecular fingerprinting technique capable of single molecular detection when ordered metallic nanostructured substrates are employed.^10^ For environmental applications, SERS measurements can be conducted with lasers in the near infrared regime where there is little interference with water^11^ and portable handheld Raman instruments enable *in situ* field measurements. One of the challenges to SERS detection is the dependency of the signal intensity on the distance from the surface (*z*), proportional to *z*^−12^, requiring analyte molecules to be within approximately *z* ≤ 4 nm for the SERS effect to be observable.^11^ Because of this requirement, SERS measurements are commonly taken from analytes dried from solution on a substrate. In an *in situ* solution phase measurement, the diffusive transport of a target analyte to the fixed surface and the adsorption affinity of the analyte are limiting factors, potentially reducing the signal strength and responsiveness of the sensor.^11,12^

Nitrate detection through normal Raman spectroscopy and SERS has been demonstrated by others.^13–21^ With advances in sensing equipment and nano-fabrication, efforts have been made to enhance detection capabilities for nutrient pollutants. Early work in the SERS field explored colloidal gold nanoparticle solutions, etched wafers, and gold sputtered nanoparticles where, for example, ions are enriched due to charge attraction and hotspots form *via* aggregation.^11,12,22^ A fixed two-dimensional substrate provides a more practical platform for continuous environmental sensing than dispersed nanoparticles.^23^*In situ* nitrate measurements were shown to be feasible at micromolar concentrations with functionalized thiol based self-assembled monolayers (SAMs) formed on commercially available substrates (LOD = 8.06 μM on Klarite™; 10 μM on Silmeco SERStrate Au)^24,25^ or with gold nanoparticles.^14,26^ Other approaches include the usage of reporter molecules on the substrate or particle surface, such as immobilized Griess reagents to form azo dyes in the presence of nitrite, which have a strong and specific Raman signal.^16,27^ For the detection of nitrate through this approach, where LODs between 1.52 μM (ref. 16) and 30.7 μM (ref. 27) nitrite have been reported, a reduction step from nitrate to nitrite is required and the diazotization is not easily reversible, limiting the feasibility for continuous in field measurement applications.^28^

An additional challenge in SERS detection is the need to overcome the diffusion barrier between solution and substrate. The application of electric fields and charged substrates has been tested to force charged analyte molecules closer to the SERS substrate.^28^ Surface functionalization with cationic ligands has been shown to increase anion adsorption on SERS sensors;^24,28,29^ however, this approach may not be feasible for field detection as the ligands are not ion selective and will attract other negatively charged organic and inorganic molecules that will interfere with the measurements. Chemometrics is a tool commonly used in the SERS community. Inclusion of advanced normalization methods and investigation of SER spectra for hidden trends through principal component analysis and machine learning approaches shows to be promising to extract formerly hidden trends from the obtained data.^30,31^

The selection of currently available techniques for *in situ* nitrate detection clearly shows the need for a modified measurement approach. Hence, we focused on non-functionalized substrates using principal component analysis, standard normal variate spectral normalization and internal silicon standards to reduce background noise and remove signal bias due to spectral features inherent to the substrates. The objective of this work is two-fold: (1) to demonstrate low level *in situ* SERS detection of nitrate in aqueous solution through statistical and internal normalization using non-functionalized nanostructured gold substrates and (2) to employ adsorption models and image charge theory to gain additional fundamental insight on nitrate detection.

## Results and discussion

### Raman analysis of dissociated NaNO_3_

Aqueous solutions of 0.25 M sodium nitrate, NaNO_3_, (Fig. S1, ESI†) were analyzed by Raman spectroscopy. The symmetric stretching mode, *ν*_1_, was identified at a wavenumber of 1047 cm^−1^ in agreement with reported values for the dissociated salt (Table 1).^32^ Close examination of the overlaid NaNO_3_ and water spectra revealed additional peaks that were common with water (bending at 1615 cm^−1^) and silicon from the glass cuvette (Fig. S2, ESI†). Less prominent peaks were also observed for both spectra near 470 cm^−1^ and 720 cm^−1^. Peaks in this region have been assigned to librational bands, *ν*_L1_ and *ν*_2_ respectively, that reflect the rotational freedom of water and intermolecular hydrogen bonding.^33–35^ The common peak ranging approximately from 1300 cm^−1^ and 1400 cm^−1^ was not precisely identified but may be due to dissolved CO_2_.^36^

**Table 1:** Sodium nitrate (NaNO_3_) structure, polarizability, hydration number, and identified Raman peaks

Formula	Anion structure	Polarizability^a^	Hydration^b^	Raman peaks^c^
NaNO_3_	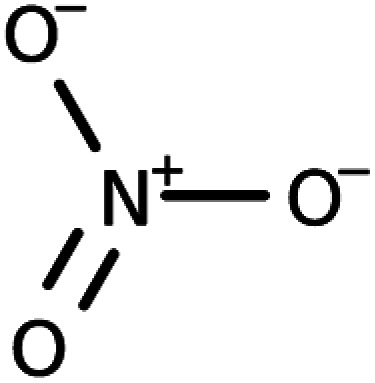	3.42	4	719 cm^−1^, bending vibration
				1047 cm^−1^, symmetric stretching

a Calculated using Marvin Sketch.

b Number of water molecules bound per anion.^37^

c Raman spectra shown in ESI (Fig. S1) (see ESI).

### SERS substrate characterization

**Fig. 1:**
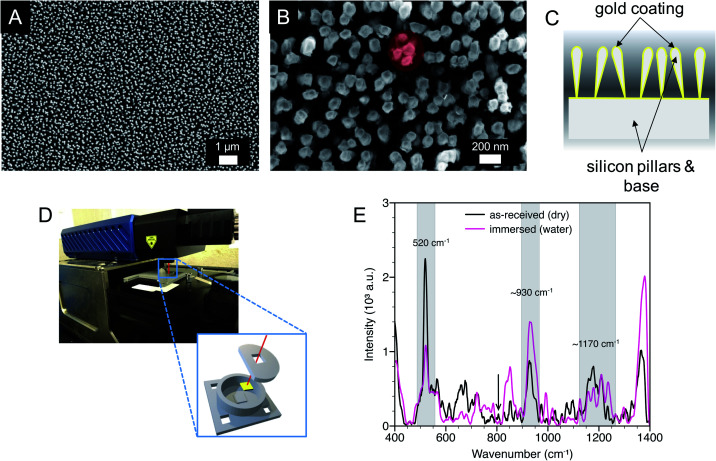
SERS substrate characterization. (A and B) SEM images of as-received substrates (top-down view of the surface; red oval shows pillar leaning to create SERS hotspots) and (C) a simplified schematic depicting the gold-on-silicon pillar configuration and partial leaning of the pillars. (D) Raman spectrometer and 3D printed ‘beaker’ to immerse the substrates. (E) SERS spectra of as-received (dry) substrates and substrates immersed in deionized water. The spectra were smoothed by a factor of four for ease of viewing. Spectra for as-received (dry) and immersed substrates were obtained with 10 s and 5 s integration times (duration of phonon detection during incident laser exposure), respectively.

The substrates were characterized by scanning electron microscopy (SEM), water contact angle measurements, and SERS. As previously reported, the substrate surfaces were homogeneous with gold-coated silicon pillars, with an effective diameter of approximately 100 nm, extending perpendicular to the substrate (Fig. 1A). Close examination revealed that the pillars were partially leaned, clustered together, and in some cases in contact, giving rise to SERS hotspots within the end region of the pillars (Fig. 1B and C).^38^ SERS measurements were conducted with a laser spot size approximately an order of magnitude larger than the size of a pillar cluster and orbital raster scanning was used with surface spot diameter of 1 to 2 mm, minimizing the effect of substrate inhomogeneities while maximizing the number of probed hot spots.

An initial water contact angle of 129° was measured on dry, as-received substrates indicative of a hydrophobic surface. After 60 min the water droplet wetted the substrate surface yielding a contact angle of 66°. When these wetted substrates were dried, and the water contact angle was again measured, there was minimal change in the initial contact angle. This observation shows that there was consistent wetting behavior of the substrates after initial water exposure. Therefore, all *in situ* SERS measurements were conducted after initial water exposure.

Raman spectra were measured for as-received (dry) SERS substrates and SERS substrates in deionized water using a 3D printed ‘beaker’ with an insert that immersed the substrate (Fig. 1D and E). Three common peaks are labeled with two peaks at approximate wavenumbers of 930 cm^−1^ and 1170 cm^−1^. The peaks centered near 930 cm^−1^ and 1170 cm^−1^ are likely due to hydrocarbons, possibly short aliphatic chains, that adsorbed from the atmosphere onto the gold surface. Additional unlabeled peaks over a wavenumber range of approximately 1280 cm^−1^ to 1400 cm^−1^ are consistent with CO_2_ and were also observed in solution Raman spectroscopy (Fig. S1, ESI†).^36^ The third peak at 520 cm^−1^ corresponds to crystalline silicon from the SERS substrate. The position and intensity of this peak when immersed in water was consistent across the substrates, providing a potential internal standard for nitrate detection. Patze *et al.*^39^ have used the silicon peak at 521 cm^−1^ arising from a similar substrate as an internal standard to improve the detection of the antibiotic sulfamethoxazole.

### Calculated ion distribution near the SERS surface

Precipitated nitrate salts are in direct contact with planar, two-dimensional SERS substrates when dried from a water droplet. This approach has shown to achieve detection limits of 0.5 mg L^−1^ (8.06 μM nitrate) using Klarite™ substrates.^25^*In situ* detection of NO_3_^−^ with immersed SERS substrates relies on the ions coming in close contact (*z* < ∼4 nm) with the substrate, or preferably the SERS hotspots.^11^ As shown by Petersen *et al.*,^40^ electrostatic interactions can be extended to polarizable metal nanostructures to determine the effects of planar dielectric discontinuity, represented as an image charge, on the distribution of ions near a metal surface. Using their approach, the self-energy arising from image charge on a planar surface (*w*_0_(*z*)) can be calculated as1
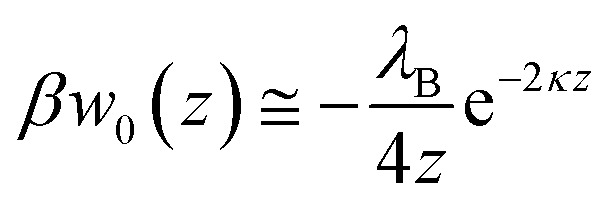
where *λ*_B_ is the Bjerrum length (0.72 nm in room temperature water), *z* is the distance from the surface to the center of the ion, and *κ* is the charge screening coefficient or square root of the inverse Debye screening length. In eqn (1), the self-energy is normalized to the thermal energy using the term *β* = (*k*_B_*T*)^−1^ where *k*_*B*_ is the Boltzmann constant and *T* is temperature. The ion distribution as a function of the distance from the surface, *n*(*z*), can be calculated from the self-energy as

2 *n*(*z*) = *n*_o_ e^−*βw*_0_(*z*)*q*_i_^2^^

where *n*_o_ is the bulk ion concentration and *q*_*i*_ is the charge of the ion (−1 for NO_3_^−^).

**Fig. 2:**
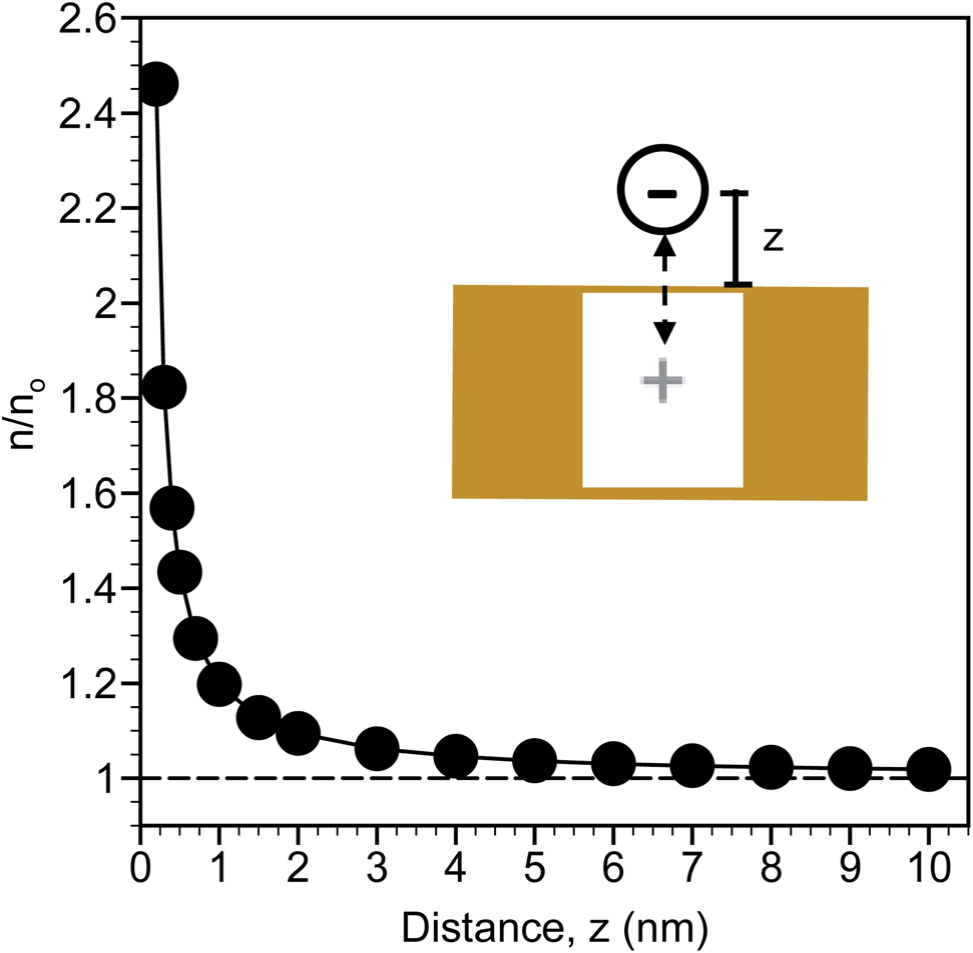
Calculated monovalent ion distribution (*n*, normalized relative to the bulk concentration as *n*/*n*_o_) extending from a planar gold surface arising from “image charge”, depicted in the schematic. The variable *z* represents the distance from the surface to the center of the ion.

The calculated distribution of monovalent ions near a planar gold surface are shown in Fig. 2. At a distance of 0.2 nm from the surface, which is slightly larger than the radii of an ion based on 0.2*λ*_B_ and approximates contact between the ion and the surface, there is nearly a 2.5-fold increase in the ion concentration compared to the bulk. The ion concentration decreases exponentially from the surface and is close to the bulk concentration at a distance of 10 nm. This analysis suggests that the region from approximately 0.2 to 3 nm can be considered an enrichment zone for SERS detection. Ion enrichment factors near the SERS surface are independent of bulk ion concentration. As a result, *in situ* SERS detection of NO_3_^−^ is expected to be a direct function of the bulk NO_3_^−^ concentration.

### *In situ* SERS detection of NO_3_^−^

**Fig. 3:**
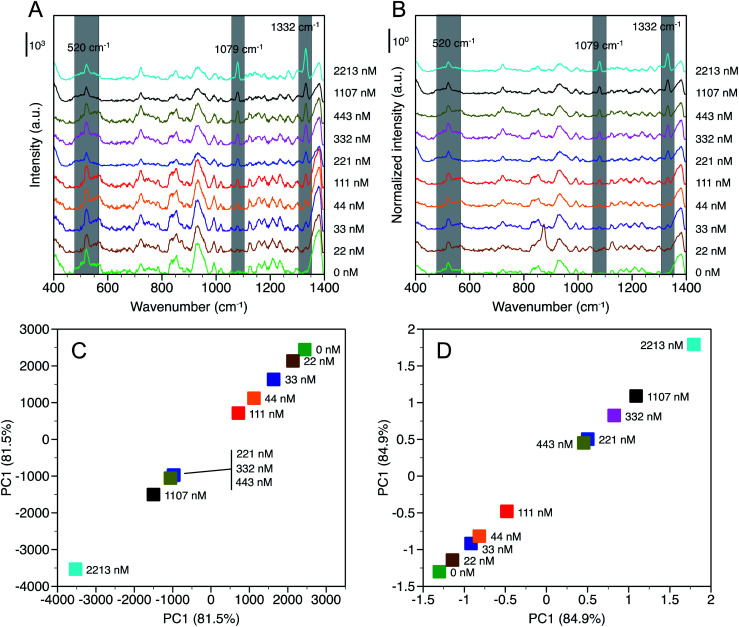
SERS spectra of nitrate based on (A) intensity (baselined) and (B) normalized intensity (baselined + SNV). Greyed regions at 1079 cm^−1^ and 1332 cm^−1^ denote NO_3_^−^ peaks used for adsorption analysis and the region at 520 cm^−1^ denotes the silicon peak from the SERS substrate. PC1 plots corresponding for (C) baselined and (D) normalized time-averaged spectra over the range 1000 cm^−1^ to 1350 cm^−1^.

SERS spectra were measured as a function of NaNO_3_ concentration, [NaNO_3_], up to 2213 nM in deionized water and are reported in terms of baselined intensity (Fig. 3A) and SNV normalized baselined intensity (Fig. 3B). Two peaks were identified at 1079 cm^−1^ and 1332 cm^−1^ that were not observed in water and that increased in intensity with [NaNO_3_]. The peak at 1079 cm^−1^ is assigned to the symmetric stretching (*ν*_1_) of NO_3_^−^ and a similar shift from 1047 cm^−1^ (observed in solution) to 1068 cm^−1^ was observed by others in the presence of chloride.^19,25^ It should be noted that the peaks observed near 930 cm^−1^ and 1170 cm^−1^ that were likely due to adsorbed hydrocarbons on the gold substrate are still present, but did not interfere with the measurement.

The origin of the peak at 1332 cm^−1^ is less clear. Gajaraj *et al.* assign dried nitrite (NO_2_^−^) to a wavenumber of 1326 cm^−1^.^25^ Elsewhere, asymmetric stretching (*ν*_3_), which is a weak Raman band compared to symmetric stretching, of NO_3_^−^ has been reported at 1345 cm^−1^ for dissociated NaNO_3_ in solution.^41^ However, we observed peaks in the same region for dissociated NaNO_3_ and water in the solution Raman spectra, which suggests this may be related to dissolved gas. In the SERS spectra (Fig. 3A and B) the peak at 1332 cm^−1^ is neighbored by a peak with a maximum near 1380 cm^−1^ that is present across the range of [NaNO_3_] examined. Based on this analysis, the 1332 cm^−1^ peak is assigned to *ν*_3_ NO_3_^−^ and the 1380 cm^−1^ peak is attributed to water or dissolved gases also observed in the solution Raman spectra.

While the NO_3_^−^ peaks identified at 1079 cm^−1^ and 1332 cm^−1^ are clearly observed the differences in peak intensity with increasing [NaNO_3_], particularly at low concentrations, are subtle. Principal component analysis (PCA) was conducted for time-averaged spectra at each [NaNO_3_] concentration over the wavenumber range from 1000 cm^−1^ to 1350 cm^−1^ to verify that these differences are statistically significant. Principal component 1 (PC1) accounted for 81.5% and 84.9% of the variance for baselined and normalized spectra, respectively. With SNV normalization, PC1 accounted for more spectral variance indicating that this method can improve NO_3_^−^ detection by accounting for spectral noise between measurements. Fig. 3C and D show that each [NaNO_3_] concentration has a distinct PC1 value, with PC1 decreasing (baselined) or increasing (normalized) with [NaNO_3_].

**Fig. 4:**
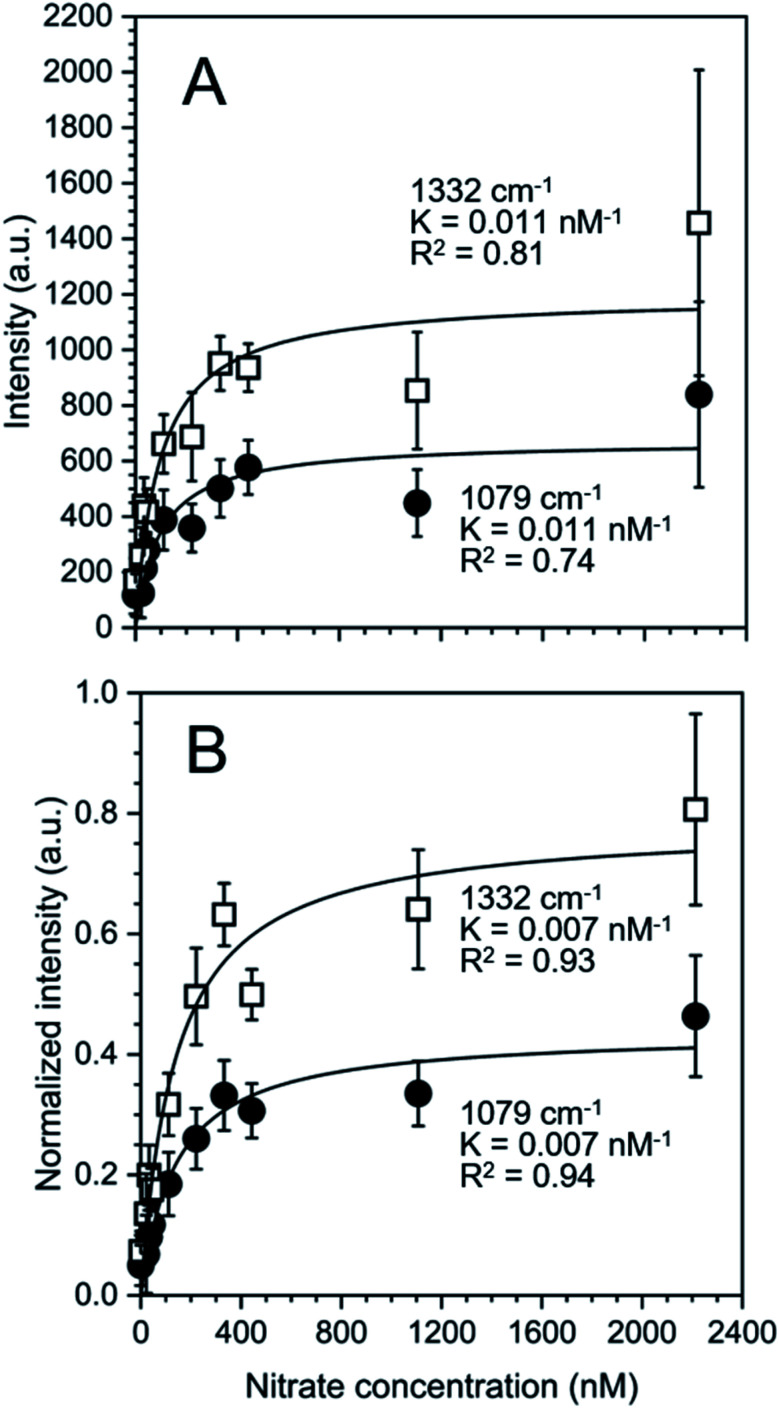
(A) Intensity and (B) normalized intensity of the peaks at 1079 cm^−1^ (closed circles ●) and 1332 cm^−1^ (open squares □) as a function of nitrate concentration. Error bars represent the time-averaged standard deviation of the intensities at the specified wavenumbers. The solid lines show the fitted Langmuir adsorption model and the Langmuir constant *K* and the goodness of fit, *R*^2^, are reported.

The nature of our measurement setup allows us to observe sorption processes in real time. We show this by plotting the time-averaged intensity and normalized intensity of SERS peaks assigned to NO_3_^−^ at 1079 cm^−1^ (*ν*_1_; Fig. 4A) and 1332 cm^−1^ (*ν*_3_; Fig. 4B) as a function of nitrate concentration. Data were analyzed with the Langmuir adsorption model assuming that the intensity or normalized intensity, *I*, were directly proportional to the concentration of NO_3_^−^ bound to the surface.3
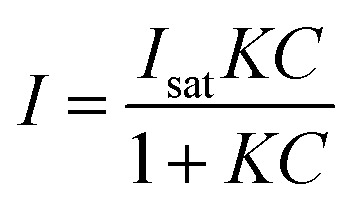


In eqn (3)*I*_sat_ is the saturated intensity or normalized intensity, *C* is the bulk [NO_3_^−^], and *K* is the Langmuir constant defined as4
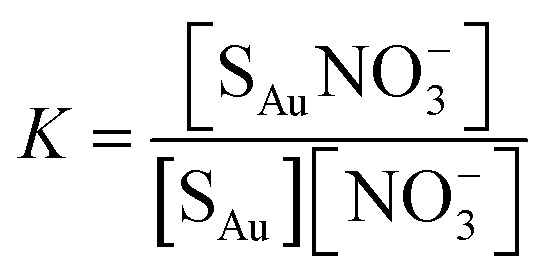
where [S_Au_] is the concentration of free gold surface binding sites and [S_Au_NO_3_^−^] is the concentration of bound sites. There was good agreement with the Langmuir model suggesting single site binding of NO_3_^−^ as a monolayer.

Based on the calculated ion distribution, this places NO_3_^−^ at the gold surface with near 2.5-fold increase in concentration relative to the bulk solution. The agreement with the Langmuir model suggests the presence of a monolayer, which is supported by the image charge theory discussed above. The *K* values associated with the model fit, ∼0.011 nM^−1^ based on intensity and ∼0.007 nM^−1^ based on normalized intensity, were the same for each peak (1079 cm^−1^ and 1332 cm^−1^), confirming that both peaks were specific to NO_3_^−^ in the SER spectra. The goodness of the fit (*R*^2^) reported in Fig. 4 shows a higher agreement of the fitting function with the SNV normalized (*R*_1079, SNV_^2^ = 0.94 and *R*_1332, SNV_^2^ = 0.93) than for the baselined data (*R*_1079, BL_^2^ = 0.74 and *R*_1332, BL_^2^ = 0.81), confirming our previous finding that SNV normalization was successfully applied to reduce spectral variability. Fitting parameters for the Langmuir model are summarized in Table S1.†

The correlation found in the data allows for the calculation of a limit of detection, by estimating the intensity at the LOD, *I*_b_, from the mean 
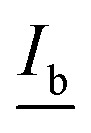
 and standard deviation *σ*_b_ of the blank^43^ according to eqn (5).5
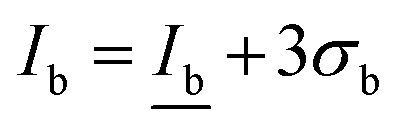


Following procedures described in the literature,^44,45^ the calculated intensity can be equated to a concentration *C*, through rearrangement of the Langmuir isotherm as shown in eqn (6).6
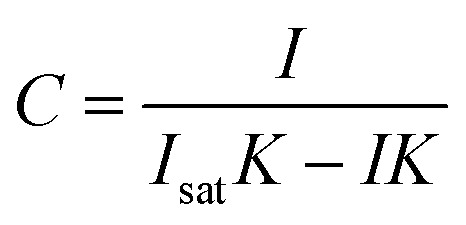


A low limit of detection of 64 nM was determined for nitrate in MilliQ water despite the measurements being conducted *in situ* with as received SERS substrates where there was no specific affinity for the target analyte.

Assuming that the Langmuir adsorption constants, *K* (Fig. 4), are equal to the equilibrium adsorption constants, the resulting adsorption free energy based on Δ*G = −RT* ln(*K*) was approximately −4 kJ mol^−1^. The adsorption entropy was determined from Δ*G* = Δ*H* − *T*Δ*S* assuming that the adsorption enthalpy, Δ*H*, can be estimated as the self-energy arising from *w*_0_(*z*) at *z* = 0.2 nm (−2.2 kJ mol^−1^). The entropy term *T*Δ*S* was 1.8 kJ mol^−1^ K^−1^ denoting a gain in entropy upon nitrate adsorption presumably due to the dehydration of the NO_3_^−^ (with a hydration number = 4; Table 1) and/or gold substrate surface.

**Fig. 5:**
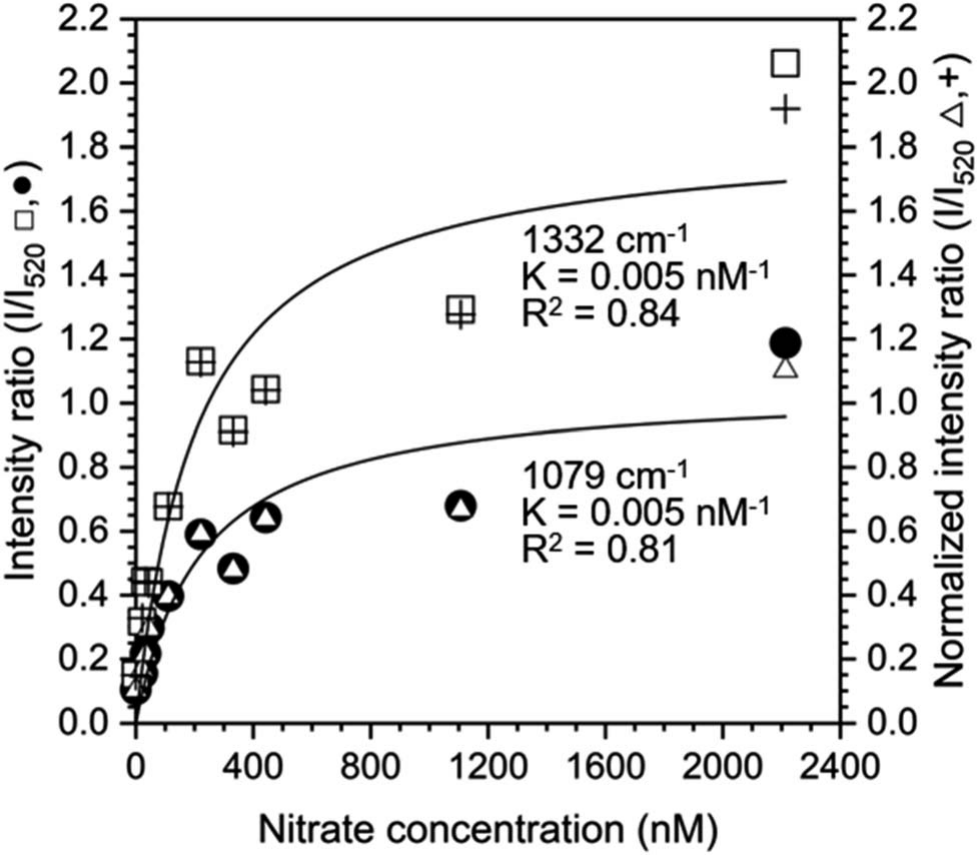
Intensity and normalized intensity ratios (*I*/*I*_520_) based on the silicon peak at 520 cm^−1^ and nitrate peaks at 1079 cm^−1^ (closed circles ●, open triangles △) and 1332 cm^−1^ (open squares □, plus signs +) as a function of nitrate concentration. The solid lines show the fitted Langmuir adsorption model and the Langmuir constant *K* and the goodness of fit, *R*^2^, are reported.

The Langmuir adsorption model was also used to fit the ratio of intensities at 1079 cm^−1^ and 1332 cm^−1^ to the intensity at 520 cm^−1^ to determine if the silicon peak is a suitable internal standard (Fig. 5). Intensity or normalized intensity ratios as a function of [NO_3_^−1^] were superimposable and the Langmuir constants, *K*, for model fits at 1079 cm^−1^ and 1332 cm^−1^ were 0.005 nM^−1^ for both peaks. These values are closer to the Langmuir constants *K* obtained from SNV normalized data (0.007 nM^−1^ for both cases) than to those of the baselined data (0.011 nM^−1^ for both cases), showing that both normalization methods yield reasonable results.

## Experimental

### Materials

Nitrate solutions were prepared by diluting sodium nitrate (NaNO_3_) standards from Sigma Aldrich (product # 53638) with ultrapure, sterile MilliQ water (resistivity of 18.2 MΩ at 25 °C). The water purity was confirmed with a Lachat Quickchem 8500 where nitrate + nitrite, if present, were below the detection limits (NO_3_^−^ + NO_2_^−^ < 250 nM). Solutions were stored in 20 mL scintillation glass vials sealed with Parafilm® at approximately 4 °C under dark conditions. Commercially available gold SERS substrates sold under the product name “SERStrate Au” were purchased from Silmeco ApS, Denmark and used as received.

### Substrate characterization

Electron micrographs of SERS substrates before and after submersion in MilliQ water were obtained with a Zeiss Sigma VP field emission scanning electron microscope (SEM) using an Everhart-Thornley secondary electron detector at an accelerating voltage of 12 kV. Previously submerged substrates were dried under ambient conditions for several hours before imaging. Drop shape analysis before and after measurements in aqueous solution was conducted with a KRÜSS DSA 100S, in sessile drop measurement mode, with a droplet volume of 2 μL MilliQ water, dispensed at a rate of 2.67 μL s^−1^, automatic baseline detection and Young Laplace fitting of the drop shape.

### Raman measurements

Normal Raman measurements, shown in the ESI,† were conducted using a SIERRA 2.0 Raman spectrometer from SnowyRange Instruments with a 785 nm laser at a power of 100 mW, a laser spot size of ∼40 μm, and an integration time of 20 s. Orbital raster scanning was used for all measurements. Confirmation of literature values of expected Raman modes was conducted by transmittance cuvette measurements of 1 mL 0.25 M nitrate in ultrapure water solution at room temperature in 3 mL glass vials. Background illumination spectra were collected and subtracted from the spectra, which were measured in triplicate and averaged for further analysis.

### SERS measurements

A top to bottom measurement configuration was used for *in situ* SERS measurements. Beaker-like devices capable of holding a volume of 0.75 mL analyte solution were 3D-printed with a MakerBot Replicator + using a poly(lactic acid) filament. The devices were used to force the SERS substrates to submerge in aqueous solution by holding them in place with a 3D-printed inset with a rectangular gap to allow for laser exposure and analyte contact. The substrate position inside the beaker was fixed due to the inset and the beaker-like device was positioned under the laser with a 3D-printed distance holder.

SERS measurements were conducted with a laser intensity of 100 mW under orbital raster scanning with an integration time of 5 s. A fresh substrate was used for every concentration series. Before the experiment was started the laser was focused by continuously measuring the Raman signal with an integration time of 0.2 s while varying the distance between substrate and laser lens until the highest signal was obtained (approximately 12 mm between laser and substrate).

Measurements were conducted with increasing concentrations of NaNO_3_ in ultrapure water, ranging from 0 to 2213 nM. A freshly 3D-printed beaker was used for each concentration series, washed with ultrapure water and dry blown with compressed nitrogen before usage. During the measurements, the instrument was covered with a housing that blocked ambient light. The background signal of the as-received, dry substrates was measured in triplicate before the beaker was filled with measurement solution. During all measurements background light subtraction was activated. Concentration series measurements were conducted by filling the beaker with MilliQ water (liquid height = 3.5 mm above substrate) and refocusing the laser to account for the focal change introduced by the medium. Measurements were started within 30 s of solvent exposure. Each concentration was measured every 2 min for up to one hour. Concentrations were increased by emptying the beaker and rinsing it with three times the beaker volume of MilliQ water, steadily dispensed from a pipette with low pressure. After the cleaning procedure, the beaker was refilled with solution of the next higher concentration. The measurement procedure was repeated until the highest concentrated solution was reached.

### Spectra analysis

Raman spectra were baselined using the TBB baseline method, a polynomial fit method implemented in the “PEAK” software distributed with the instrument, at a sensitivity of 115 out of 1000. Spectra were also normalized by the standard normal variate method^42^ according to the following equation7
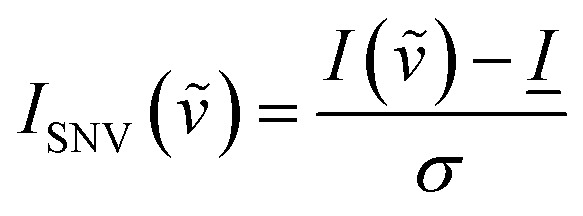
where *I*_SNV_(*ν̃*) and *I*(*ν̃*) are the SNV modified and unmodified peak intensities, respectively, at a given wavenumber *ν̃*, *I̲* is the average intensity of the spectrum, and *σ* is the standard deviation of the spectrum. The method produced negative intensity values of the baseline on the order of −0.4 a.u. for all spectra. To allow for the comparison of normalized intensities by a common starting point, and to allow for easier fitting the baseline for all spectra was manually shifted to zero, so that no negative values remained in the spectra.

Principal Component Analysis (PCA) was applied to all collected spectra after baselining with and without the application of SNV. To conduct PCA, spectra were grouped by concentration and analyzed with the OriginPro application “PCA for Spectroscopy” (Version 2019b, OriginLab Corporation, Northampton MA, USA).

## Conclusions

In this work we developed a method for the *in situ* SERS detection of nitrate dissolved in ultrapure water, using as received nanostructured gold substrates, without further modification. Applying electrophysical theory to our system we argue that an NO_3_^−^ ion enrichment zone close to the gold substrate is present. *In situ* detection of nitrate was shown to be possible with low limits of detection in the nanomolar regime. Langmuir adsorption behavior for increasing nitrate concentrations averaged from multiple time points, was identified using our method. Three different data processing techniques (baselining without normalization, SNV normalization, and normalization by an internal standard) were evaluated, showing that both normalization methods improve the accuracy of our results. Our work shows the potential of SERS, not only for the detection of environmental pollutants in complex media, but also for studying the interactions of such pollutants with surfaces in the marine environment. The universal detection capabilities of Raman spectroscopy, as well as the superposition of spectral information, suggest that such a sensor could be extended to detect multiple analytes simultaneously and to other compounds containing nitro groups such as pesticides or explosives. Further work is required to increase the signal strength and yield more robust sensors for field applications.

## Conflicts of interest

There are no conflicts to declare.

## Supplementary Material

NA-003-D1NA00156F-s001
