# Supplementary material for: In situ SERS detection of dissolved nitrate on hydrated gold substrates
Source: Nanoscale Adv. 2021 Jun 12;3(14):4098–105. doi: 10.1039/d1na00156f (PMC9418535; doi:10.1039/d1na00156f)
Supplement: NA-003-D1NA00156F-s001 [file NA-003-D1NA00156F-s001.pdf]

Electronic Supplementary Material (ESI) for Nanoscale Advances.
This journal is © The Royal Society of Chemistry 2021

Electronic Supporting Information (ESI)

In Situ SERS detection of dissolved nitrate on hydrated gold substrates

Timo Küster and Geoffrey D. Bothun*

Department of Chemical Engineering, University of Rhode Island, 2 East Alumni Ave, Kingston, RI, 02881 USA

Corresponding author: gbothun@uri.edu, +1-401-874-9518

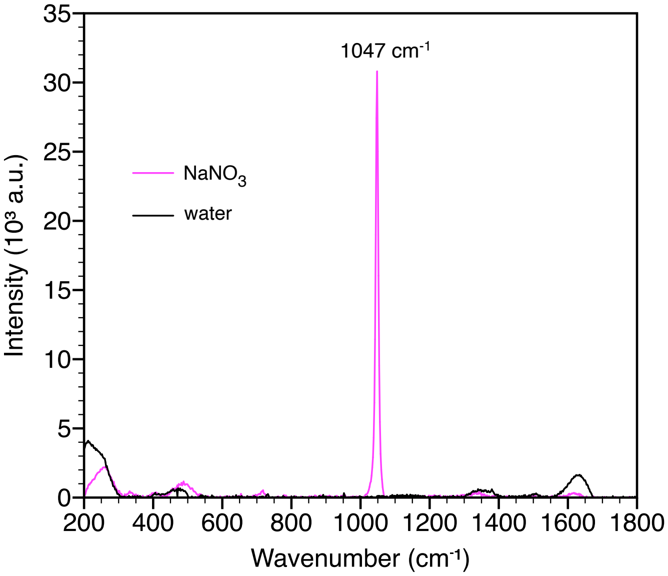

Figure S1. Raman spectra of 0.25 M $NaNO_3$ measured at room temperature with integration time of 20 s and at a laser power of 100 mW. The peak corresponding to symmetric stretching (ν_1) was identified at 1047 cm^{-1} in agreement with William and Begun[1] for 1 M $NaNO_3$ at 25 ^{o}C.

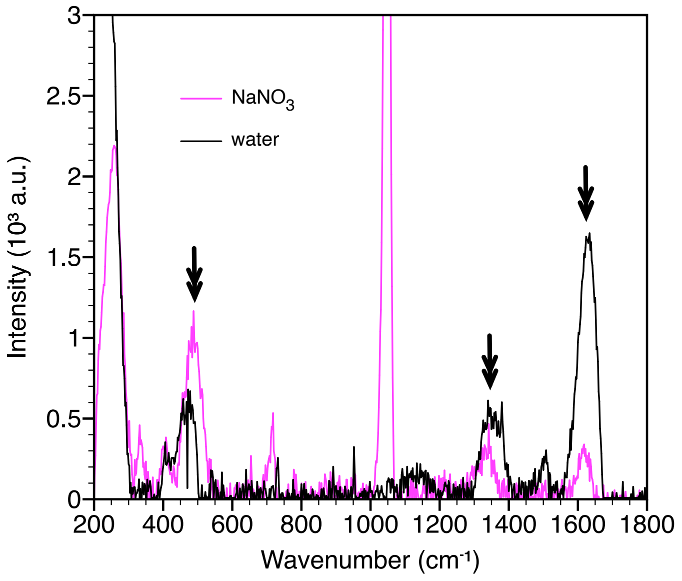

Figure S2. Magnified Raman spectra shown in Figure S1. The peak at 480 cm^{-1} with a shoulder peak near 520 cm^{-1} reflect a mixture of amorphous and crystalline silicon, respectively, from the glass cuvette. The peak at 1615 cm^{-1} is assigned to water bending. An additional unassigned peak is observed near 1330 cm^{-1} in both spectra.

Calculations

Table S1: Fitting parameters derived for Langmuir adsorption model. K represents the Langmuir constant and I_{sat} is the intensity at the saturation concentration. The subscripts indicate the wavenumbers at which the data was evaluated.

Processing method	K_{1079} (nM^{-1})	$I_{sat, 1079}$	R^2_{1079}	K_{1332} (nM^{-1})	$I_{sat, 1332}$	R^2_{1332}
**Baselined**	0.0105	672.3	0.74	0.0106	1194.7	0.81
**SNV**	0.0071	0.4369	0.94	0.0072	0.7829	0.93
**Si normalization (baselined)**	0.0045	1.0530	0.81	0.0047	1.8537	0.84
**Si normalization (SNV)**	0.0057	0.9591	0.82	0.0059	1.705	0.85

The limit of detection was calculated for the SNV normalized data by calculating the intensity at the LOD (equation 5 in manuscript)

$$I_b = \underline{I_b} + 3 \cdot \sigma_b = 0.0500 + 3 * 0.0289 = 0.1367$$

and substituting I with I_b in the rearranged Langmuir equation (equation 6 in manuscript) to obtain the limit of detection concentration.

$$C = \frac{I}{I_{sat} \cdot K - I \cdot K} = \frac{0.1367}{0.4369 \cdot 0.0071\, nM^{-1} - 0.1367 \cdot 0.0071\, nM^{-1}} = 64.1\, nM$$

References

1 W. L. Marshall and G. M. Begun, *J. Chem. Soc., Faraday Trans. 2*, 1989, **85**, 1963–1978.
